# Cytotoxycity and antiplasmodial activity of phenolic derivatives from *Albizia zygia* (DC.) J.F. Macbr. (Mimosaceae)

**DOI:** 10.1186/s12906-019-2792-1

**Published:** 2020-01-15

**Authors:** Romeol Romain Koagne, Frederick Annang, Bastien Cautain, Jesús Martín, Guiomar Pérez-Moreno, Gabin Thierry M. Bitchagno, Dolores González-Pacanowska, Francisca Vicente, Ingrid Konga Simo, Fernando Reyes, Pierre Tane

**Affiliations:** 1 Department of Chemistry, Faculty of Science, University of Dschang, P.O. Box 67, Dschang, Cameroon; 2 Fundación MEDINA, Centro de Excelencia en Investigación de MedicamentosInnovadores en Andalucía, Avda. delConocimiento 34, Parque Tecnológico de Ciencias de la Salud, E-18016 Granada, Spain; 3 Instituto de Parasitología y Biomedicina “López-Neyra”, Consejo Superior de Investigaciones Científicas (CSIC) Avda. del Conocimiento s/n, 18016, Armilla, Granada, Spain

**Keywords:** Phenolic compounds, Anticancer activity, *Plasmodium falciparum*, *Albizia zygia*

## Abstract

**Background:**

The proliferation and resistance of microorganisms area serious threat against humankind and the search for new therapeutics is needed. The present report describes the antiplasmodial and anticancer activities of samples isolated from the methanol extract of *Albizia zygia* (Mimosaseae).

**Material:**

The plant extract was prepared by maceration in methanol. Standard chromatographic, HPLC and spectroscopic methods were used to isolate and identify six compounds (**1–6**). The acetylated derivatives (**7–10**) were prepared by modifying 2-*O*-*β*-D-glucopyranosyl-4-hydroxyphenylacetic acid and quercetin 3-*O*-*α*-L-rhamnopyranoside, previously isolated from *A. zygia* (Mimosaceae). A two-fold serial micro-dilution method was used to determine the IC_50s_ against five tumor cell lines and *Plasmodium falciparum*.

**Results:**

In general, compounds showed moderate activity against the human pancreatic carcinoma cell line MiaPaca-2 (10 < IC_50_ < 20 μM) and weak activity against other tumor cell lines such as lung (A-549), hepatocarcinoma (HepG2) and human breast adenocarcinoma (MCF-7and A2058) (IC_50_ > 20 μM). Additionally, the two semi-synthetic derivatives of quercetin 3-*O*-*α*-L-rhamnopyranoside exhibited significant activity against *P. falciparum* with IC_50_ of 7.47 ± 0.25 μM for compound **9** and 6.77 ± 0.25 μM for compound **10**, higher than that of their natural precursor (IC_50_ 25.1 ± 0.25 μM).

**Conclusion:**

The results of this study clearly suggest that, the appropriate introduction of acetyl groups into some flavonoids could lead to more useful derivatives for the development of an antiplasmodial agent.

## Background

*Albizia* is a large genus belonging to the Mimosaceae plant family. It comprises at least 150 species mostly trees and shrubs native to tropical and subtropical regions of Asia and Africa [1]. In traditional medicine, the roots bark of *Albizia zygia* are used against cough, while its stem bark is used as a purgative, antiseptic, aphrodisiac, to treat gastritis, fever, conjunctivitis, as well as to fight worms and overcome female sterility [2, 3]. The methanol extract of its stem bark has been reported to exhibit strong activity against *P. falciparum* K1 strain and *Trypanosoma brucei rhodesiense* [4–6]. The genus *Albizia* is phytochemically known as a source of saponin compounds with a large number of sugar moieties [3, 7, 8]. Despite this predisposition to produce saponins, previous works have also reported flavonoids, alkaloids and tannins [9–11]. Thus, we carried out and reported herein the fractionation and purification of methanol extract of *A. zygia* followed by the acetylation of the two most abundant isolated compounds obtained, 2-*O*-*β*-D-glucopyranosyl-4-hydroxyphenylacetic acid and quercetin 3-*O*-*α*-L-rhamnopyranoside. The cytotoxic and antiplasmodial activities of compounds are also reported.

## Methods

### General experimental procedures

Column chromatography were proceeded with Silica gel 60 *F*_254_ (70–230; Merck; Darmstadt, Germany). TLC developed on precoated silica gel Kieselgel 60 *F*_254_ plates (0.25 mm thick) and compounds were detected by spraying with 50% H_2_SO_4_ on it before being heated at 100 °C. Semi-preparative and preparative HPLC was performed using a Gilson FX-281322H2 High Performance Liquid Chromatography coupled to a DAD detector and an automatic fraction collector. ASunfire C18 column (10 μm, 10 × 250 mm) and (5 μm, 10 × 150 mm) were used in these separations. (+)-ESITOF-MS was performed as previously described [12]. We recorded NMR spectra on a Bruker Avance III spectrometer, equipped with a 1.7 mm TCI microcryoprobe, (500.0 and 125.0 MHz for ^1^H and ^13^C NMR, respectively). The chemical shifts are given in part per million (ppm) using the signal of the residual solvent as internal reference. The coupling constant (*J*) are in Hertz.

### Plant material

The leaves of *Albizia zygia* (DC) J.F. Macbr were collected on the slopes of the cliff of Santchou, West Region of Cameroon in March 2013. It is a public and well known wild. Thus, access and collection of samples do not require any permission according to the legislation of Cameroon. These leaves were identified at the National Herbarium of Cameroun (NHC) by comparison to a voucher specimen under the number N° 43,969 HNC.

### Extraction and isolation

Dried leaves of *A. zygia* were ground to a fine powder (0.77 Kg) and macerated with methanol (5 L) for 24 h (repeated 3 times) at room temperature. After filtration and removal of the solvent in vacuo, a crude extract of 42.0 g was obtained. The extract was subjected to silica gel column chromatography (CC) eluting with gradient of *n*-hexane-EtOAc and then EtOAc-MeOH to afford four major fractions (A-D). Fraction A was not further investigated, it contains mostly fatty material and fraction B (3.2 g) was separated by column chromatography over silica gel with a (5–30%) of *n*-hexane-EtOAc to give quercetin (**6**) (27.0 mg). Fraction C (12.6 g) was separated by column chromatography over silica gel using gradient (5–50%) of CH_2_Cl_2_-MeOH to give a mixture of compounds **2** and **3** (97.3 mg). Fraction D (20.8 g) was subjected to silica gel column chromatography eluted with gradient (5–40%) of EtOAc-MeOH to give phaseoloidin (**1**) (335.6 mg) and a mixture of **4** and **5** (9.8 mg). Further purification of the two above mentioned mixtures by semi-preparative HPLC eluted with a gradient of acetonitrile-water from 5 to 100% as mobile phase, afforded quercetin 3-*O*-*α*-L-rhamnopyranoside (**2**) (44.4 mg) and kampherol 3-*O*-*α*-L-rhamnopyranoside (**3**) (13.7 mg) from the first mixture, and quercetin 3,4′-di-*O*-*α*-L-rhamnopyranoside (**4**) (1.6 mg) and kaempferol 3,4′-di-*O*-*α*-L-rhamnopyranoside (**5**) (1.1 mg) from the second one.

### Semi-synthetic compounds

***Acetylation of 2-O-β-D-glucopyranosyl-4-hydroxyphenylacetic acid (1)***: 2-*O*-*β*-D-glucopyranosyl-4-hydroxyphenylacetic acid (10.0 mg, 3.03 10^− 5^ mol) was dissolved in 1 mL of pyridine, 0.25 mL of acetic anhydride (0.026 mol) were added, and the mixture was left to stand for 24 h. Extraction with CH_2_Cl_2_ and semi-preparative HPLC purification (ACN-H_2_O, 5–100) gave two new derivatives: compounds **7** (2.2 mg, yield:15%) and **8** (1.9 mg, yield: 11%).

***2-O-β-D-glucopyranosyl-4-hydroxyphenylacetic acid (1):*** white powder;^1^H NMR (500 MHz, DMSO-*d*_*6*_): *δ*_*H*_ 6.60 (d, *J* = 2.6 Hz, H-3), 6.57 (dd, *J* = 2.6 and 8.7 Hz, H-5), 6.95 (d, *J* = 8.7 Hz, H-6), 3.58 (s, H-7), 4.53 (d, *J* = 6.7 Hz, H-1′), 3.51 (d, *J* = 16.5 Hz, H-2′), 3.67 (d, *J* = 11.9 Hz, H-3′), 3.61 (d, *J* = 15.9 Hz, H-4′), 3.13 (m, H-5′), 3.45 (m, H-6′); ^13^C NMR (125 MHz, DMSO-*d*_*6*_): *δ*_*C*_ 173.7 (C-8), 35.6 (C-7), 117.6 (C-4), 117.6 (C-5), 118.0 (C-3), 126.6 (C-1), 152.7 (C-2), 103.3 (C-1′), 73.9 (C-2′), 77.0 (C-3 ‘), 70.3 (C-4’), 77.5 (C-5 ‘), 61.5 (C-6’); (+)-HRESI-MS: *m/z* 348.1288 (calcd. For C_14_H_22_O_9_N, 348.1289).

***Compound 7:*** colorless oil; ^1^H NMR (500 MHz, MeOD):*δ*_*H*_ 7.01(d, *J* = 2.6 Hz, H-3), 6.65 (dd, *J* = 8.6 and 2.6 Hz, H-5), 6.69 (d, *J* = 2.6 Hz, H-6), 3.62 (d, *J* = 16.4 Hz, H-7*α*), 3.46 (d, *J* = 16.4 Hz, H-7*β*), 5.35 (t, *J* = 7.4 Hz, H-1′), 4.33 (dd, *J* = 5.0 and 12.2 Hz, H-2′), 5.13 (m, H-3′), 4.18 (dd, *J* = 2.6 and 12.3 Hz, H-4′), 3.99 (m, H-5′), 5.17 (m, H-6’*α*), 5.11 (m, H-6’*β*), 2.10 (s, 3H), 2.08 (s, 3H), 2.05 (s, 3H), 2.01 (s, 3H); HRESI-MS (+): *m/z *516.1708 (calcd for C_22_H_30_NO_13,_ 516.1712).

***Compound 8***: colorless oil; ^1^H NMR (500 MHz, MeOD):* δ*_*H*_ 7.01 (d, *J* = 2.4 Hz, H-3), 6.99 (dd, *J* = 8.9 and 2.4 Hz, H-5), 7.17 (d, *J* = 8.9 Hz, H-6), 3.68 (d, *J* = 15.0 Hz, H-7), 3.48 (d, *J* = 15.9 Hz, H-7), 5.29 (d, *J* = 7.3 Hz, H-1′), 4.34 (dd, *J* = 5.5 and 12.3 Hz, H-2′), 5.21 (*J* = 2.1 and 7.5 Hz, H-3′), 4.17 (dd, *J* = 2.4 and 12.3 Hz, H-4′), 4.08 (m, H-5′), 5.16 (m, H-6’*α*), 5.12(m, H-6’*β*), 2.09 (s, 3H), 2.07 (s, 3H), 2.04 (s, 3H), 2.01(s, 3H), 2.26 (s, 3H); HRESI-MS (+): *m/z* 558.1814 (calcd for C_24_H_32_NO_14_, 558.1817).

***Acetylation of quercetin 3-O-α-L-rhamnyranoside (2)*** Quercetin 3-*O*-*α*-L-rhamnyranoside (22.0 mg, 4.91 10^− 5^ mol) was dissolved in 2.5 mL of pyridine, and 0.75 mL of acetic anhydride (0.0079 mol) were added, the mixture was left to stand for 24 h. Extraction with CH_2_Cl_2_ and semi-preparative HPLC purification gave two new derivatives: compounds **9** (7.6 mg, yield 18%) and **10** (2.8 mg, yield 6%).

***Quercetin 3-O-α-L-rhamnyranoside (2):*** yellow powder; ^1^H NMR (500 MHz, MeOD): *δ*_*H*_ 6.32 (s, H-6), 6.17 (s, H-8), 7.35 (s, H-2′), 7.29 (d, *J* = 7.9 Hz, H-6′), 6.92 (d, *J* = 7.9 Hz, H-5′), 5.36 (s, H-1″), 3.79 (d, *J* = 8.8 Hz, H-2″), 3.44 (m, H-3″), 3.37 (m, H-4″), 4.26 (m, H-5″), 0.91 (d, *J* = 6.1 Hz, H-6″); ^13^C NMR (125 MHz, MeOD): *δ*_C_ 134.8 (C-3), 178.1 (C-4), 156.9 (C-5), 93.5 (C-6), 164.7 (C-7), 98.6 (C-8), 157.9 (C-9), 104.3 (C-10), 121.6 (C-1′), 115.7 (C-2′), 144.9 (C-3′), 148.4 (C-4′), 115.1 (C-5′), 121.7 (C-6′), 102.2 (C-1″), 70.8 (C-2″), 70.6 (C-3″), 71.9 (C-4″), 70.5 (C-5″), 16.3 (C-6″); (+)-HRESI-MS: *m/z* 449.1076 (calcd. 449.1078 for C_21_H_21_O_11_).

***Compound 9***: yellow oil; ^1^H NMR (500 MHz, MeOD): *δ*_*H*_ 6.23 (d, *J* = 1.9 Hz, H-6), 6.41 (d, *J* = 1.9 Hz, H-8), 7.35 (d, *J* = 2.2 Hz, H-2′), 6.96 (d, *J* = 7.1 Hz, H-5′), 7.33 (dd, *J* = 2.2 and 7.1 Hz, H-6′), 5.60 (d, *J* = 1.6 Hz, H-1″), 5.63 (d, *J* = 3.3 Hz, H-2″), 5.28 (d, *J* = 3.3 Hz, H-3″), 4.88 (m, H-4″), 3.41 (m, H-5″), 0.87 (d, *J* = 6.3 Hz, H-6″), 2.13 (s, 11-Me), 2.02 (s, 13-Me), 1.99 (s, 15-Me); ^13^C NMR (125 MHz, MeOD): *δ*_*C*_ 133.1 (C-3), 161.9 (C-5), 93.3 (C-6), 164.1 (C-7), 98.6 (C-8), 157.2 (C-9), 104.5 (C-10), 120.9 (C-1′), 121.4 (C-2′), 145.4 (C-3′), 148.6 (C-4′), 114.9 (C-5′), 115.2 (C-6′), 97.8 (C-1″), 68.7 (C-2″), 69.2 (C-3″), 70.0 (C-4″), 68.1 (C-5″), 16.1 (C-6″), 170.0 (C-11), 18.9 (C-12), 170.6 (C-13), 19.2 (C-14), 170.3 (C-15), 19.0 (C-16); (+)-HRESI-MS: *m/z* 575.1388 (calcd. 575.1395 for C_27_H_27_O_14_).

***Compound 10***: yellow oil; ^1^H NMR (500 MHz, MeOD): *δ*_*H*_ 6.56 (d, *J* = 2.3 Hz, H-6), 6.82 (d, *J* = 2.5 Hz, H-8), 7.33 (d, *J* = 2.1 Hz, H-2′), 6.96 (d, *J* = 7.7 Hz, H-5′), 7.32 (dd, *J* = 2.0 and 7.1 Hz, H-6′), 5.46 (d, *J* = 1.3 Hz, H-1″), 5.29 (d, *J* = 3.6 Hz, H-2″), 5.27 (d, *J* = 3.6 Hz, H-3″), 4.77 (m, H-4″), 3.37 (m, H-5″), 0.87 (d, *J* = 6.1 Hz, H-6″), 2.13 (s, 11-Me), 2.02 (s, 13-Me), 1.98 (s, 15-Me), 2.37 (s, 17-Me); ^13^C NMR (125 MHz, MeOD): *δ*_*C*_ 133.1 (C-3), 161.9 (C-5), 108.7 (C-6), 163.8 (C-7), 100.3 (C-8), 157.2 (C-9), 104.5 (C-10), 120.9 (C-1′), 115.1 (C-2′), 145.4 (C-3′), 148.6 (C-4′), 114.9 (C-5′), 121.4 (C-6′), 97.9 (C-1″), 68.7 (C-2″), 69.2 (C-3″), 70.0 (C-4″), 68.1 (C-5″), 15.9 (C-6″), 170.0 (C-11), 19.1 (C-12), 170.4 (C-13), 19.0 (C-14), 170.3 (C-15), 19.0 (C-16), 169.9 (C-17), 19.5 (C-18); (+)-HRESI-MS**:** *m/z* 617.1497 (calcd for C_29_H_29_O_15,_ 617.1501).

***P. falciparum 3D7 lactate dehydrogenase assay***: Parasites of the *P. falciparum* strain 3D7 were grown in fresh group 0 positive human erythrocytes, obtained from the Centro Regional de Transfusion Sanguınea-SAS (Granada, Spain). This assay was performed in duplicate for each compound using a sixteen (16) point dose response curve (½ serial dilutions) with concentrations starting from 50 μM until 1.5 nM to determine the IC_50_s of the compounds. Adding 25 μL of *P. falciparum* 3D7 parasite culture (per well) containing parasitized red blood cells at 0.25% parasitaemia and 2% haematocrit in RPMI-1640, 5% Albumax II, 2% D-sucrose 0.3% glutamine and 150 μM hypoxanthine and incubated at 37 °C for 72 h with 5% CO_2_, 5% O_2_ and 95% N_2_. For negative and positive growth controls, 10 μM chloroquine and complete parasite growth medium were respectively used. The final readouts of the assay was done by measuring the absorbance of the reactions at 650 nm in an Envision plate reader (Perkin Elmer, USA) and the results analysed by Genedata software (GenedataAG, Basel, Switzerland), parasite growth was measured by LDH assay as previously described [12, 13].

***Anticancer assays:*** Five tumor cell lines (MiaPaca-2 (CRL-1420), a carcinoma pancreatic from 65 years adult; Hep G2 (HB-80665), a perpetual cell line which was derived from the liver tissue of a 15-year-old Caucasian American male with a well-differentiated hepatocellular carcinoma; A549 (CCL-185), a carcinoma lung from 58-year-old Caucasian made; A2058 (CRL-11147), Human skin melanoma from a 43 years Caucasian adult derived from lymph node and MCF-7 (HTB-22), a breast adenocarcinoma from 69 years woman) were obtained from ATCC. The MTT (3-(4,5-dimethylthiazol-2-yl)-2,5-diphenyltetrazoliumbromide) colorimetric assay, which measures mitochondrial metabolic activity, was employed to estimate the amount of living cells. According to the huge amount of celles to be plated, SelecT (TAP Biosystems, Royston, UK), a cell culture robotic system was used to process ten thousand cells per well (for 72 h assay). Cells were seeded at a concentration of 1× 104 cells/well in 200 μl culture medium and incubated at 37 °C in 5% CO_2_. After 24 h, the automated liquid-handling system Biomek FX (Beckman Coulter, Pasadena, CA, USA) was used to replace the medium with a final volume of 200 μL and 1 μL of compound (dilution 1/200) and to add controls to the plates and which were then be incubated for 72 h. The test compounds were examined in triplicate with serial two-fold dilutions. After incubation, MTT solution was prepared at 5 mg/mL in PBS 1X and then diluted at 0.5 mg/mL in MEM without phenol red. The sample solution in wells was removed and 100 μL of MTT dye was added to each well. The plates were gently shaken and incubated for 3 h at 37 °C in 5% CO_2_ incubator. The supernatant was removed and 100 μL of DMSO 100% was added. The plates were gently shaken to solubilize theoriginated formazan and absorbance at 570 nm was read in a Victor2 Wallac spectrofluorometer (PerkinElmer, Waltham, MA, USA). IC_50_ values were calculated as the concentration that decreases 50% of the cell viability using Genedata Screener software (Genedata AG, Basel, Switzerland). Curve fitting followed the Smart Fit strategy with Hill model selection.

## Results

The methanol extract of the leaves of *A. zygia* was purified over silica gel, Sephadex LH-20 column chromatography and HPLC to afford six phenolic compounds (**1**–**6**); two of them were subjected to acetylation to give four new semi-synthetic compounds. The structures of the isolated compounds were determined by spectroscopic and spectrometric data and comparison with those of similar reported compounds. Both, naturally occurring and semi-synthetically prepared metabolites were screened for their antiplasmodial and cytotoxic properties.

### Phytochemical analysis

**Fig. 1:**
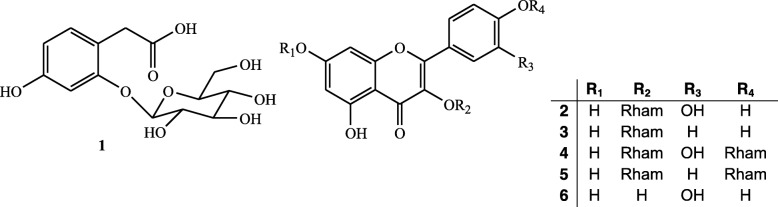
Chemical structure of compounds isolated from *A. zygia*1–6

The natural occurring compounds were already described in the literature, phaseoloidin (**1**), quercetin 3-*O*-*α*-L-rhamnopyranoside (**2**), kaempferol 3-*O*-*α*-L-rhamnopyranoside (**3**), quercetin 3,4′-di-*O*-*α*-L-rhamnopyranoside (**4**), kaempferol 3,4′-di-*O*-*α*-L-rhamnopyranoside (**5**) and quercetine (**6**) (Fig. 1) [14–16]. Phaseoloidin was previously reported from the *Nicotiana attenuate* trichomes [14] and this is the first report of its occurrence in the genus *Albizia*. On the contrary, all the isolated flavonoids have been previously obtained from other species of *Albizia* genus.

### Chemical transformation

**Fig. 2:**
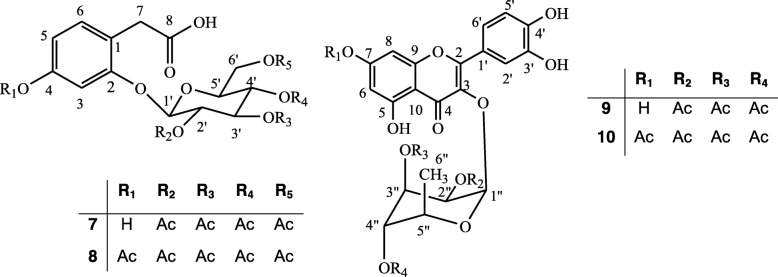
Chemical structure of new semi synthetic compounds **7**–**10**

The starting materials, 2-*O*-*β*-D-glucopyranosyl-4-hydroxyphenylacetic acid and quercetin 3-*O*-*α*-L-rhamnopyranoside, isolated from the leaves of *A. zygia,* were subjected to acetylation by reacting with acetic anhydride in pyridine, followed by semi-preparative HPLC purification. The structures of the semi-synthetic derivatives **7**–**10** (Fig. 2) were determined on the basis of their NMR and HRESI-MS data and comparison with those of compounds **1** and **2**.

**Compound 7** was obtained as colorless oil with a molecular formula of C_22_H_26_O_13_ deduced from its (+)-ESI-TOF-MS which showed an ammonium adduct [M + NH_4_]^+^ at *m/z* 516.1708 (calcd. 516.1712 for C_22_H_30_NO_13_). Its structure was deduced by comparing its ^1^H NMR data with those of 2-*O*-*β*-D-glucopyranosyl-4-hydroxyphenylacetic acid (**1**). Indeed, the ^1^H NMR spectrum of **7** exhibited signals of three aromatic protons at *δ*_*H*_ 7.01 (d, 1H, *J* = 8.6 Hz, H-6), 6.69 (d, 1H, *J* = 2.6 Hz, H-3) and 6.65 (dd, 1H, *J* = 8.6 and 2.6 Hz, H-4) and two methylene protons at *δ*_*H*_ 3.62 (d, 1H, *J* = 16.4 Hz, H-7*α*) and 3.46 (d, 1H, *J* = 16.4 Hz, H-7*β*). In addition to these signals common to **1**, the spectrum displayed signals of four methyl groups at *δ*_*H*_2.10 (s, 3H), 2.08 (s, 3H), 2.04 (s, 3H) and 2.01 (s, 3H), corresponding to methyl protons of four aliphatic acetyl groups, indicating the acetylation of the four free hydroxyl groups of the glucose moiety of **1**. Aliphatic hydroxyl groups, like those of the sugar moiety, are more reactive than those of the phenol groups [17, 18].

**Compound 8** was obtained as colorless oil. A molecular formula of C_24_H_28_O_14_ was deduced from its (+)-ESI-TOF-MS which showed an ammonium adduct [M + NH_4_]^+^ at *m/z* 558.1814 (calcd. 558.1817 for C_24_H_32_NO_14_). As for compounds **1** and **7**, the ^1^H NMR spectrum displayed three aromatic protons at *δ*_*H*_ 7.17 (d, 1H, *J* = 8.9 Hz, H-6), 7.01 (d, 1H, *J* = 2.4 Hz, H-3) and 6.69 (dd, 1H, *J* = 8.9 and 2.4 Hz, H-5) and a methylene group at *δ*_*H*_ 3.68 (d, 1H, *J* = 15.0 Hz, H-7*α*) and 3.48 (d, 1H, *J* = 15.0 Hz, H-7*β*). Four methyl groups were also observed at *δ*_*H*_ 2.09 (s, 3H), 2.07 (s, 3H), 2.04 (s, 3H) and 2.01 (s, 3H) corresponding to the acetylated sugar moiety. Additionally, the spectrum showed the signal of a fifth methyl group attributable to the aromatic acetyl at *δ*_*H*_ 2.26 (s, 3H) confirming the peracetylation of compound **1**.

**Compound 9** was obtained as yellow oil. The molecular formula C_27_H_26_O_14_ was deduced from its positive mode (+)-ESI-TOF-MS, which showed a pseudo-molecular ion [M + H]^+^ at *m/z* 575.1388 (calcd. 575.1395 for C_27_H_27_O_14_). Its structure was deduced from that of quercetin 3-*O*-*α*-L-rhamnoside (**2**). In fact, the ^1^H NMR spectrum of **9** exhibited signals characteristics of the B ring at *δ*_*H*_ 7.35 (d, 1H, *J* = 2.2 Hz), 7.33 (dd, 1H, *J* = 2.2 and 7.1 Hz) and 6.96 (d, 1H, *J* = 7.1 Hz) assignable to H-2′, H-6′ and H-5′, respectively. Additionally, signals of the A ring at *δ*_*H*_ 6.41 (d, 1H, *J* = 1.9 Hz) and 6.23 (d, 1H, *J* = 1.9 Hz), assigned to H-8 and H-6, respectively, were also observed. The anomeric proton at *δ*_*H*_ 5.60 (d, 1H, *J* = 1.6 Hz, H-1″), the signals of methine groups at *δ*_*H*_ 5.30 (d, 1H, *J* = 3.3 Hz, H-2″), 5.28 (d, 1H, *J* = 3.3 Hz, H-3″), 3.43 (m, 1H, H-4″) and 3.41 (m, 1H, H-5″) and the methyl group at 0.87 (d, 3H, *J* = 6.3 Hz, H-6″) recalled those signals of a rhamnose moiety in the structure of **9**. In addition to these signals common to compound **2**, the spectrum also showed three methyl groups at *δ*_*H*_ 1.99 (s, 3H), 2.02 (s, 3H) and 2.13 (s, 3H) corresponding to three acetyl groups. The HMBC spectrum revealed that these methyls were located on the sugar moiety.

**Compound 10** was obtained as yellow amorphous powder. Its molecular formula, C_29_H_28_O_15_, was assigned from its positive mode (+)-ESI-TOF-MS, which showed a pseudo-molecular ion [M + H]^+^ at *m/z* 617.1493 (calcd. 617.1501 for C_29_H_29_O_15_). The ^1^H NMR spectrum of compound **10** displayed signal patterns similar to those of compounds **2** and **9**, including the three protons of B ring at *δ*_*H*_ 7.33 (d, 1H, *J* = 2.1 Hz, H-2′), 7.32 (dd, 1H, *J* = 2.1 and 8.7 Hz, H-6′) and 6.96 (d, 1H, *J* = 8.7 Hz, H-5′) and the two protons of A ring at *δ*_*H*_ 6.82 (d, 1H, *J* = 2.5 Hz, H-8) and 6.56 (d, 1H, *J* = 2.5 Hz, H-6), assignable to the flavonoid part of the molecule. In addition to signals corresponding to the three acetyl groups already observed in compound **9** at *δ*_*H*_ 1.98 (s, 3H), 2.02 (s, 3H) and 2.13 (s, 3H), the spectrum showed an additional methyl group attributable to an aromatic acetyl group at *δ*_*H*_ 2.37 (s, 3H) linked to C-7. One can noticed the deshielding of signals from carbons C-8 and C-6 compared to their homolog compounds **9** and **2**. The fact that only the hydroxyl at C-7 was acetylated can be explained also by the chelation observed between the hydroxyl group at C-5 and the carbonyl at C-4 and between the two hydroxyl groups at C-3′ and C-4′, which will make the latter hydroxyl groups less reactive than the OH-7. Appropriate NMR and MS spectra are provide as supplementary material (Additional file 1: fig. S1 - fig. S14).

### Antiplasmodial activity

The natural compounds isolated from the leaves of *A. zygia* as well as their semi-synthetic derivatives were tested against *Plasmodium falciparum* (Table 1) using a microdilution method in liquid medium as previously described [13]. The two semi-synthetic derivatives of quercetin 3-*O*-*α*-L-rhamnopyranoside exhibited significant activity against *P. falciparum* with IC_50_ values of 7.5 ± 0.25 μM for compound **9** and 6.8 ± 0.25 μM for compound **10**. However, the natural precursor of these two semi-synthetic derivatives showed a weak activity (IC_50_ 25.1 ± 0.25 μM), similar to that of kaempferol 3-*O*-*α*-L-rhamnopyranoside (**3**) (IC_50_ 19.0 ± 0.25 μM). The natural precursor 2-*O*-*β*-D-glucopyranosyl-4-hydroxyphenylacetic acid (**1**) and its semi-synthetic derivatives **7** and **8** together with quercetin 3,4′-di-*O*-*α*-L-rhamnopyranoside (**4**) and kaempferol 3,4′-di-*O*-*α*-L-rhamnopyranoside (**5**) did not show any activity against *P. falciparum* (IC_50_ > 100 μM). Chloroquine gave an IC_50_ of 2.96 ± 0.25 nM when tested under the same conditions.

**Table 1:** IC_50_ of natural and semi-synthetics compounds from *A. zygia* against *P. falciparum*

	Compounds IC_50_ (SI) in μM									
	1	2	3	4	5	7	8	9	10	Chloroquine
*P. falciparum*	> 100	25.1 ± 0.25	19.0 ± 0.25	> 100	> 100	> 100	> 100	7.5 ± 0.25	6.8 ± 0.25	0.00296
	(nd)	(3.49)	(1.22)	(nd)	(nd)	(nd)	(nd)	(3.03)	(9.57)	–

SI = IC_50_ HepG2 cell/IC_50_ *P. falciparum*

### Anticancer activity

The natural compounds **1**–**5** as well as the semi-synthetic derivatives **7**–**10**, were screened for cytotoxic effects against five human tumor cell lines namely MiaPaca-2 (pancreas), A-549 (lung), HepG2 (liver), MCF-7 (breast) and A2058 (breast) (Table 2). The compounds showed moderate activity against MiaPaca-2 with IC_50_ values of 17.3 ± 0.25, 16.8 ± 0.25, 10.0 ± 0.25, 18.5 ± 0.25 and 17.4 ± 0.25 μM for quercetin 3,4′-di-*O*-*α*-L-rhamnopyranoside (**4**), kaempferol 3,4′-di-*O*-*α*-L-rhamnopyranoside (**5**), compounds **7**, **8** and **9**, respectively. Compound **9** also showed moderate activity against MCF-7 (IC_50_ 10.8 ± 0.25 μM) and A-2058 (IC_50_ 12.2 ± 0.25 μM) as well as quercetin 3,4′-di-*O*-*α*-L-rhamnopyranoside (**4**) against MCF-7 IC_50_ (17.3 ± 0.25 μM) and HepG2 (IC_50_ 17.3 ± 0.25 μM). According to the screening program of the National Cancer Institute, USA, a compound is generally considered to have in vitro cytotoxic activity if the IC_50_ value following incubation between 48 and 72 h, is less than 4 μg/mL or 10 μM [19]. In the present report, IC_50_ values below or around this threshold (10 μM) were obtained with compound **9** against MCF-7 (IC_50_ 10.8 μM) and compound **7** against Miapaca-2 (IC_50_ 10.0 μM).

**Table 2:** Cytotoxycity of natural and semi-synthetics compounds from *A. zygia*

Cell lines	Compounds IC_50_ (μM)									
	1	2	3	4	5	7	8	9	10	Doxorubicin
MCF-7	42.7 ± 0.25	87.5 ± 0.25	46.4 ± 0.25	17.3 ± 0.25	33.7 ± 0.25	37.0 ± 0.25	40.2 ± 0.25	10.8 ± 0.25	64.9 ± 0.25	< 7 × 10^−5^
A2058	66.7 ± 0.25	87.5 ± 0.25	46.4 ± 0.25	34.6 ± 0.25	33.7 ± 0.25	37.0 ± 0.25	40.2 ± 0.25	12.2 ± 0.25	64.9 ± 0.25	< 7 × 10^−5^
HepG2	121.2 ± 0.25	87.5 ± 0.25	23.2 ± 0.25	17.3 ± 0.25	16.8 ± 0.25	37.0 ± 0.25	40.2 ± 0.25	22.6 ± 0.25	64.9 ± 0.25	< 7 × 10^−5^
A-549	121.2 ± 0.25	89.5 ± 0.25	23.2 ± 0.25	34.6 ± 0.25	33.7 ± 0.25	20.1 ± 0.25	20.1 ± 0.25	34.8 ± 0.25	30.5 ± 0.25	< 7 × 10^−5^
MiaPaca-2	30.3 ± 0.25	87.5 ± 0.25	46.4 ± 0.25	17.3 ± 0.25	16.8 ± 0.25	10.0 ± 0.25	18.5 ± 0.25	17.4 ± 0.25	64.9 ± 0.25	< 7 × 10^−5^

## Discussion

The genus *Albizia* is so far a source of natural occurring saponins and phenolics [3, 7, 8, 20, 21]. In our study, no saponins were isolated but phenolic compounds were obtained. Chemical composition of plants can differ from one species to another in a group of plants. That can be due to the ecological region where plants are growing. However, this experiment allowed us to confirm once more that *Albizia* genus continues to be a source of polar compounds as our phenolics were glycosylated. This study aimed also at identifying how acetylation of phenolic compounds can interfere with the antiplasmodial and anticancer activities by comparing IC_50_ values of precursors to those of semi-synthetic compounds. The results indicate that acetylated derivatives display in general a better activity than their natural precursors.

The antiplasmodial activities of the isolated compounds were 19–100.0 μM and that of acetylated derivatives were 6.8–100.0 μM against *Plamodium falciparum* strain 3D7. Derivatives **9** (7.5 μM) and **10** (6.8 μM) scored the highest in vitro activity among the compounds tested. Several flavonoids have been reported to exert a moderate antiplasmodial activity in a number of different *P. falciparum* strains [22–24]. As a result, we present herein a difference in activity of high hydroxylated flavonoids compared to their acetylated derivatives. This result is interesting insofar that acetylation reaction is easy to achieve in laboratories and flavonoids are very common in plants. Thus, the appropriate introduction of acetyl groups into flavonoids may lead to more useful derivatives for the development of an antiplasmodial agent. In fact, the two acetylated compounds **9** and **10** were over 3 times more active than their natural precursor quercetin 3-*O*-*α*-L-rhamnopyranoside (**2**). However, the absence of activity of phaseolidin (**1**) and its corresponding derivatives **7** and **8** highlighted that hydroxyl groups are not related to the absence of activity of compound **1** on the protozoal *P. falciparum*. This is the first report of the antiplasmodial activity of the 2-*O*-*β*-D-glucopyranosyl-4-hydroxyphenylacetic acid and quercetin 3-*O*-*α*-L-rhamnopyranoside derivatives.

On the other hand and according to the screening program of the National Cancer Institute, USA, a compound is generally considered to have in vitro cytotoxic activity if it exhibits an IC_50_ ≤ 4.0 mg/mL or 10.0 μM, following its incubation for 48 and 72 h with cancer cells [19]. In the present report, IC_50_ values equal or around this threshold (10.0 μM) were obtained with compounds **10** (10.8 and 12.2 μM against MCF-7and A2050 respectively) and **7** (10.0 μM against Miapaca-2). In general, as shown in Table 2, the lowest IC_50_ were obtained with the semisynthetic derivatives (IC_50_ 10.0–64.9 μM) compared to the parent compounds (IC_50_ 16.8–121.2 μM). The current result is in the same line with those previously described in the literature which shows that flavonoids have good anticancer properties [25, 26]. All the compounds isolated and described in this report could be said to be generally non-cytotoxic when compared to the standard drug Doxorubicin which showed an IC_50_ ≈ 0.0 μM.

However, the theoretical more effectivity and safety of our compounds was calculated. Compound **10** presented a better safety capability (SI = 9.57) compared to its counterpart compound **9** (SI = 3.03). For the others, the toxicity of the drugs was not far enough from the antiplasmodial effects (SI < 3) to guarantee their useness. The toxicity of the flavonoids could be said to be related to the hydroxyl group at C-7.

## Conclusion

The objective of this study was to highlight the effect of structure transformation through acetylation of phenolic compounds over anticancer and antiplasmodial activities. The results clearly suggest that, the appropriate introduction of acetyl groups into flavonoids may lead to more useful derivatives for the development of antiplasmodial and anticancer agents.

## Supplementary information

**Additional file 1.** Supplementary Informations, **Figure S1 - Figure S14**.

## Data Availability

All data generated or analysed during this study are included in this published article and its supplementary information files.

## Abbreviations

ABC: ATP-binding cassette
BCRP: Breast cancer resistance protein
D.R.: Resistance
DMSO: Dimethylsulfoxide
EGFR: Epidermal growth factor receptor
FITC: Flouresceinisothiocynate
H2DCFH-DA: 2′,7′-dichlorodihydrofluoresceine diacetate
H_2_O_2_: Hydrogen peroxide
JC-1: 5,5′,6,6′-tetrachloro-1,1′,3,3′-tetraethylbenzimidazolylcarbocyanine iodide
IC_50_: 50% inhibitory concentration
MDR: Multidrug resistance
MMP: Mitochondrial membrane potential
M-PERs: Mammalian Protein Extraction Reagent
PBS: Phosphate buffer saline
PARP-1: Poly (ADP-ribose) polymerase 1
P-gp: P-glycoprotein
PI: Propidium iodide
RIP-3: Receptor-interacting protein 3
ROS: Reactive oxygen species
RT: Room temperature
SDS–PAGE: Sodium dodecyl sulfate-polyacrylamide gel electrophoresis

## References

[1] Karuppannan K, Subramanian DP, Venugopal S (2013). Phytopharmacological properties of *Albizia* species: a review. Int J Pharm Pharm Sci.

[2] Abdalla MA, Laatsch H. Flavonoids from Sudanese *Albizia zygia* (Leguminosae, subfamily mimosoideae), a plant with antimalarial potency. Afr J Tradit Complement Altern Med. 2012;9:56–8. 10.4314/ajtcam.v9i1.8 PMC3746526 23983320

[3] Note OP, Chabert P, Pegnyem DE, Weniger B, Dubois ML, Lobstei A (2010). Structure elucidation of new acacic acid-type saponins from *Albizia coriaria*. Magn Reson Chem.

[4] Kigondu EVM, Rukunga GM, Keriko JM, Tonui WK, Gathirwa JW, Kirira PG, Irungu B, Ingonga JM, NdiegeI O (2009). Anti-parasitic activity and cytotoxicity of selected medicinal plants from Kenya. J Ethnopharmacol.

[5] Wanyama PAG, Kiremire BT, Murumu JES, Kamoga O (2011). Textile dyeing and phytochemical characterization of crude plant extracts derived from selected dye-yielding plants in Uganda. Int J Nat Prod Res.

[6] Lenta BN, Vonthron-Sénécheau C, Soh RF, Tantangmo F, Ngouela S, Kaiser M, Tsamo E, Anton R, Weniger B (2007). In vitro antiprotozoal activities and cytotoxicity of some selected Cameroonian medicinal plants. J Ethnopharmacol.

[7] Cheng ZQ, Yang D, Ma QY, Yi XH, Zhang NL (2011). Triterpenoid saponins from *Albizia mollis*. Bul Korean Chem.

[8] Singab AN, Bahgat D, Al-Sayed E, Eldahshan O (2015). Saponins from genus *Albizia*: phytochemical and biological review. J Med Arom.

[9] Gupta RS, Chaudhary R, Yadav RK, Verma SK, Dobhal MP (2005). Effect of saponins of *Albizia lebbeck* (L.) Benth bark on the reproductive system of male albino rats. J Ethnopharmacol.

[10] Abdel-Kader M, Hoch J, Berger JM, Evans R, Miller JS (2000). Two bioactive saponins from *Albizia subdimidiata* from the Suriname rainforest. J Nat Prod.

[11] Debella A, Haslinger E, Schmid MG, Bucar F, Michl G (2001). Triterpenoid saponins and sapogenin lactones from *Albizia gummifera*. Phytochemistry.

[12] Martín J, Crespo G, González-Menéndez V, Pérez-Moreno G, Sánchez-Carrasco P, Pérez-Victoria I, Ruiz-Pérez LM, González-Packanowska D, Vicente F, Genilloud O, Bills GF, Reyes F (2014). MDN-0104, an antiplasmodial betaine lipidfrom *Heterosporachenopodii*. J NatProd.

[13] Pérez-Moreno G, Cantizani J, Sánchez-Carrasco P, Ruiz-Pérez LM, Martín J, El Aouad N, Pérez-Victoria I, Tormo JR, González-Menendez V, González I, de Pedro N, Reyes F, Genilloud O, Vicente F, González-Pacanowska D (2016). Discovery of new compounds active against *Plasmodium falciparum*by high throughput screening of microbial natural products. PLoSOne.

[14] Weinhold A, Wenzler M, Schneider B, Baldwin IT (2011). Phaseoloidin, a homogentisic acid glucoside from *Nicotiana attenuata* trichomes, contributes to the plant's resistance against lepidopteran herbivores. J Chem Ecol.

[15] Marzouk MS, El-Toumy SAA, Merfort I, Nawwar MAM (1999). Polyphenolic metabolites of Rhamnusdisperma. Phytochemistry.

[16] Shaheen F, Ali L, Ali S (2009). ErdemogluN, Sener B. antioxidant flavonoids from *Tamus communis* ssp. Critica. J Chem Nat Compd.

[17] Ardhaoui M, Falcimaigne A, Engasser JM, Moussou P, Pauly G, Ghoul M (2004). Acylation of natural flavonoids using lipase of *candida antarctica* as biocatalyst. J Mol Catal B Enzym.

[18] Chebil L, Anthoni J, Humeau C, Gerardin C, Engasser JM, Ghoul M (2007). Enzymatic acylation of flavonoids: effect of the nature of the substrate, origin of lipase, and operating conditions on conversion yield and regioselectivity. J Agric Food Chem.

[19] Brahemi G, Kona FR, Fiasella A, Buac D, Soukupova J, Brancale A, Burger AM, Westwell AD (2010). Exploring the structural requirements for inhibition of the ubiquitin E3 ligase breast cancer associated protein 2 (BCA2) as a treatment for breast cancer. J Med Chem.

[20] Rukunga GM, Waterman PG (1996). (1996) Kaempferol glycosides from *Albizia versicolor*. Bull Chem Soc Ethiop.

[21] Lau CS, Carrier DJ, Beitle RR, Bransby DI, Howard LR, Lay JJO, Liyanage R, Clausen EC (2007). Identification and quantification of glycoside flavonoids in the energy crop *Albizia julibrissin*. Bioresour Technol.

[22] Gopiesh KV, Kannabiran K, Rajakumar G, Rahuman AA, Santhoshkumar T (2011). Biolarvicidal compound gymnemagenol isolated from leaf extract of miracle fruit plant, *Gymnemasylvestre* (Retz) Schult against malaria and filariasis vectors. Parasitol Res.

[23] Kraft C, Jenett-Siems K, Siems K, Gupta MP, Bienzle U, Eich E (2000). Antiplasmodial activity of isoflavones from *Andirainermis*. J Ethnopharmacol.

[24] Lehane AM, Saliba KJ (2008). Common dietary flavonoids inhibit the growth of the intraerythrocytic malaria parasite. BMC Res Notes.

[25] Kuete V, Sandjo LP, Kwamou GMN, Wiench B, Nkengfack AE, Efferth T (2014). Activity of three cytotoxic isoflavonoids from *Erythrina excelsa* and *Erythrina senegalensis* (neobavaisoflavone, sigmoidin H and isoneorautenol) toward multi-factorial drug resistant cancer cells. Phytomedecine.

[26] Li H, Zhang X, Wang W (2017). Anticancer activity of 5,7-dimethoxyflavone against liver cancer cell line HepG2 involves apoptosis, ros generation and cell cycle arrest. Afr J Tradit Complement Altern Med.

