# Supplementary material for: Cytotoxycity and antiplasmodial activity of phenolic derivatives from Albizia zygia (DC.) J.F. Macbr. (Mimosaceae)
Source: BMC Complement Med Ther. 2020 Jan 15;20:8. doi: 10.1186/s12906-019-2792-1 (PMC7076739; doi:10.1186/s12906-019-2792-1)
Supplement: Supplementary file 1 — Additional file 1. Supplementary Informations, Figure S1 - Figure S14. [file 12906_2019_2792_MOESM1_ESM.docx]

**SUPPORTING INFORMATIONS FILE**

**Title: Cytitoxycity and antimalarial activity of phenolic derivatives from *Albizia zygia* (DC.) J.F. Macbr. (Mimosaceae)**

Romeol Romain Koagne^a,b^, Frederick Annang^b^, Bastien Cautain^b^, Jesús Martín^b^, Guiomar Pérez-Moreno^c^, Gabin Thierry M. Bitchagno^a^, Dolores González-Pacanowska^c^**,** Francisca Vicente^b^, Ingrid Simo Konga^a^*, Fernando Reyes^b^, Pierre Tane^a^*

*^a^ Department of Chemistry, Faculty of Science, University of Dschang, P.O. Box 67, Dschang, Cameroon.*

*^b^ Fundación MEDINA, Centro de Excelencia en Investigación de Medicamentos Innovadores en Andalucía, Avda. delConocimiento 34, Parque Tecnológico de Ciencias de la Salud, E-18016 Granada, Spain.*

*^c^Instituto de Parasitología y Biomedicina "López-Neyra", Consejo Superior de Investigaciones Científicas (CSIC) Avda. del Conocimiento s/n, 18016, Armilla, Granada, Spain.*

***Corresponding authors**

Ingrid Simo Konga : [simoingrid@yahoo.fr](mailto:ingridsimo@yahoo.fr); Tel : (+237) 677748776; *P.O. Box 67 Dschang, Cameroon*

Pierre Tane : E-mail: ptane@yahoo.com; Tel: +237 677-61-95-46; *P.O. Box 67 Dschang, Cameroon*

**Abstract**

**Background:** The proliferation and resistance of microorganisms are a serious threat against humankind and the search for new therapeutics is needed. The present report describes the antimalarial and anticancer activities of samples isolated from the methanol extract from *Albizia zygia* (Mimosaseae).

**Material:** The plant extract was prepared by maceration in methanol. Standard chromatographic, HPLC and spectroscopic methods were used to isolate and identify six compounds (**1-6**).The acetylated derivatives (**7-10**) were prepared by modifying of 2-*O*-*β*-D-glucopyranosyl-4-hydroxyphenylacetic acid and quercetin 3-*O*-*α*-L-rhamnopyranoside, previously isolated from *A. zygia* (Mimosaceae). A three-fold serial micro-dilution method was used to determine the IC_50s_ against five tumour cell lines and *Plasmodium falciparum*.

**Results:** In general, compounds showed moderate activity against the human pancreatic carcinoma cell line MiaPaca-2 (10 ˂ IC_50_ ˂ 20 μM) and weak activity against other tumor cell lines such as lung (A-549), hepatocarcinoma (HepG2), human breast adenocarcinoma (MCF-7and A2058) (IC_50_ ˃ 20 μM). Additionally, the two semi-synthetic derivatives of quercetin 3-*O*-*α*-L-rhamnopyranoside exhibited significant activity against *P. falciparum* with IC_50_ of 7.47 ± 0.25 μM for compound **9** and 6.77 ± 0.25 μM for compound **10**, higher than that of their natural precursor (IC_50_ 25.10 ± 0.25μM).

**Conclusion:** The results of this study clearly suggest that, the appropriate introduction of acetyl groups into flavonoids may lead to more useful derivatives for the construction of an antimalarial agent.

**Keywords:** Phenolic compounds, anticancer activity, *Plasmodium falciparum*, *Albizia zygia*.

**CONTENTS**

**Figure S1:** Positive HRESI-MS spectrum of compound **7**

**Figure S2:**  ^1^H NMR spectrum (MeOD, 500 MHz) of compound **7**

**Figure S3:** Positive HRESI-MS spectrum of compound **8**

**Figure S4:** ^1^H NMR spectrum (MeOD, 500 MHz) of compound **8**

**Figure S5:** Positive HRESI-MS spectrum of compound **9**

**Figure S6:**  ^1^H NMR spectrum (MeOD, 500 MHz) of compound **9**

**Figure S7:** COSY (^1^H-^1^H) spectrum of Compound **9**

**Figure S8:** HSQC spectrum of compound **9**

**Figure S9:** HMBC spectrum of compound **9**

**Figure S10:** Positive HRESI-MS spectrum of compound **10**

**Figure S11:** ^1^H NMR spectrum (MeOD, 500 MHz) of compound **10**

**Figure S12:** COSY (^1^H-^1^H) spectrum of Compound **10**

**Figure S13:** HSQC spectrum of compound **10**

**Figure S14:** HMBC spectrum of compound **10**

**
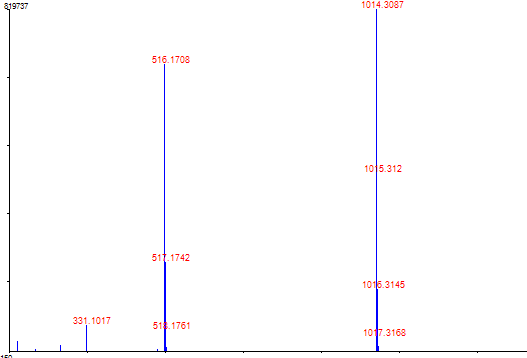
**

[M+NH_4_]^+^

**Figure S1: Positive HRESI-MS spectrum of compound 7**

H-1’

4 x CH_3_

H-5

H-7

H-6

H-3

**Figure S2: ^1^H NMR spectrum (MeOD, 500 MHz) of compound 7**

**
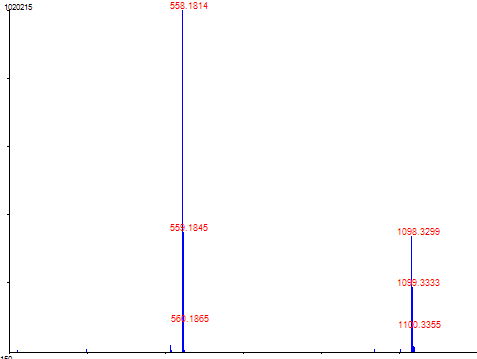
**

[M+NH_4_]^+^

**Figure S3: Positive HRESI-MS spectrum of compound 8**

5 x CH_3_

H-1’

H-6

H-3

H-5

H-7

**Figure S4: ^1^H NMR spectrum (MeOD, 500 MHz) of compound 8**

**
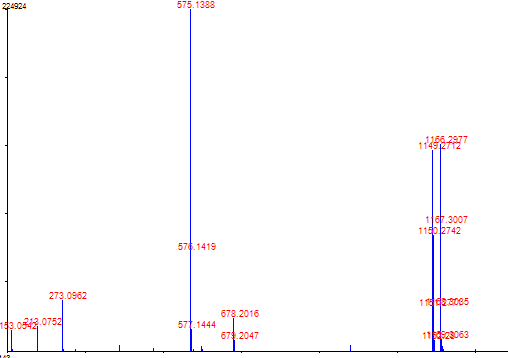
**

[M+H]^+^

**Figure S5: Positive HRESI-MS spectrum of compound 9**

3 x CH_3_

H-2’

H-6’

H-8

H-6

H-5’

H-1’’

H-6’’

**Figure S6: ^1^H NMR spectrum (MeOD, 500 MHz) of compound 9**

**Figure S7: COSY (^1^H-^1^H) spectrum of Compound 9**

**Figure S8: HSQC spectrum of Compound 9**

**Figure S9: HMBC spectrum of Compound 9**

**
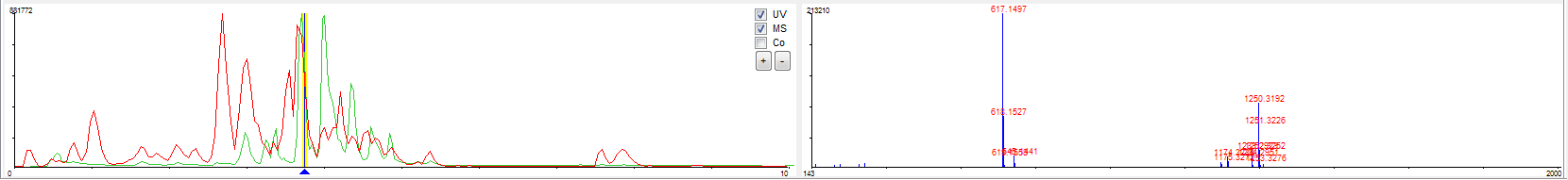
**

[M+H]^+^

**Figure S10: Positive HRESI-MS spectrum of compound 10**

4 x CH_3_

H-2’

H-1’’

H-8

H-6

H-5’

H-6’

H-6’’

**Figure S11: ^1^H NMR spectrum (MeOD, 500 MHz) of compound 10**

**Figure S12: COSY (^1^H-^1^H) spectrum of Compound 10**

**Figure S13: HSQC spectrum of Compound 10**

**Figure S15: HMBC spectrum of Compound 10**
